# “I feel like it should be but I know it’s really not”: exploring physical fitness priorities at the correctional training program in Canadian federal corrections

**DOI:** 10.1080/17482631.2024.2375662

**Published:** 2024-07-02

**Authors:** Zachary Towns, Rosemary Ricciardelli, Dale C. Spencer

**Affiliations:** a Department of Maritime Studies, Public Safety, Security, and Wellness, Marine Institute, Memorial University of Newfoundland, St. John’s, Newfoundland & Labrador, Canada; b Research Chair in Public Safety, Security, and Wellness, Department of Maritime Studies, Marine Institute, Memorial University of Newfoundland, St. John’s, Newfoundland & Labrador, Canada; c Faculty of Public Affairs Research Excellence Chair, Department of Law and Legal Studies, Carleton University, Ottawa, Ontario, Canada

**Keywords:** Prison work, correctional officer, body work, correctional services, Canada

## Abstract

The correctional training program (CTP), composed of three stages, includes a 14 week in-person component that Correctional Officer Recruits (CORs) must complete prior to their employment as a federal Correctional Officer (CO) for Correctional Service Canada (CSC). The CTP prepares recruits for a plethora of CO responsibilities, some dependent on physical fitness, such as responding to codes being called, physical altercations, or violent situations. Unlike other public safety positions (e.g. policing, border services, or coast guard) CSC does not require occupational fitness testing. In the current article, we use data from a multiyear longitudinal study of federal COs from across Canada to unpack how CORs manage physical fitness expectations at CTP; CSCs’ expectations of COR physical fitness; and outline what types of physical fitness (e.g. weightlifting, cardiovascular, self-defence) are taught, thus valued most, during CTP. We situate the voices of CORs regarding physical fitness within the broader “body” literature and discuss policy recommendations tied to physical fitness, specifically COs’ interest in reinstating pre-employment physical fitness screening.

## Introduction

Correctional Officers (COs) are tasked with supervising, supporting, managing, caring for, and providing essential services to prison residents (Griffin et al., 2012; Turner et al., 2023). Dowden and Tellier (2004) argue the balance between care, control, and institutional security are sources of stress for COs, who manage an array of roles and responsibilities (Crawley, 2004, Leibling et al., 2010; Dowden & Tellier, 2001; Weinrath, 2017). Within these roles, the bodily aptitude requirements for the CO occupation receives little scholarly attention, despite the interconnection between occupational needs, physical ability, and how exercise programs positively impact mental health (Battagalia et al., 2014; Pastor & Cucci, 2018). Evidence within the extant CO physical fitness scholarship suggests that COs experience unfavourable working conditions tied to understaffing, exposure to violence, burnout, negative relationships with colleagues (i.e., gossip), critical incidents (stabbings, grievous death or bodily harm, suicides) or boredom (see Boudoukha et al., 2011; Cassiano & Ricciardelli, 2023; Johnston et al., 2021; Spencer et al., in press; Viotti, 2016). Their physicality, here, may also serve as a protective factor for the potential adverse consequences of correctional service provision.

In response, in the current study, we unpack data from a multi-year longitudinal study of federal Correctional officer recruits (CORs) (*n* = 41) to understand the value they place on physical fitness, as a recruitment requirement method (i.e., or lack thereof), during the correctional training program (CTP), and once deployed into a penitentiary. We studied how recruits are exposed to fitness expectations and physically demanding training exercises (e.g., self-defence) during CTP and unpack the tension between the lack of occupational fitness testing and the demands on their physicality for the profession. Our intention is to begin a conversation about how physical fitness prepares COs for prison work, and how (if at all) physical fitness practices are carried over when deployed to a penitentiary.

In this article we unpack baseline data from CORs, prior to their occupational start date at a federal Canadian penitentiary to better understand the value placed on physical fitness at the CTP. First, we review existing literature on reflexive body techniques, corporal schema, and existing forms of physical fitness in prisons to better situate our findings. We then outline our methods, mainly a semi-grounded emergent theme approach to 41 semi structured interviews with recruits elucidating their responses relating to physical fitness. Our results are structured based on three emergent themes all relating to physical fitness: How CORs interpret fitness at the CTP, how CORs perceive the fitness levels of fellow recruits during CTP, and how physical fitness is enforced during the CTP. We conclude with a discussion on how the required phenomenological schema and reflexive body techniques of correctional work require CORs to be physically fit and how occupational fitness standards and the potential tools necessary for CORs to be fit are largely missing. Ending with a discussion of ways forward for physical fitness practices and potential policy changes for federal correctional services in Canada.

## Bodies, physical fitness, and reflexive body techniques

Writing in 1934, Emile Durkheim’s nephew, Mauss (1973) argued culture was best explained through ‘techniques of the body.’ He began by asserting that humans first and most natural instrument is their bodies. As such, he directs his analysis towards body techniques and thereby, denaturalizes the taken-for-granted practices making up everyday life. In addition, body techniques are evaluated in terms of their efficiency in relation to training and the acquisition of additional skills and efficiency.

Drawing from Mauss, Crossley (2005) introduces the concept of *reflexive body techniques* (RBT). RBTs are “those body techniques whose primary purpose is to work back upon the body, so as to modify, maintain or thematize it in some way” (Crossley, 2005, p. 9). RBTs may involve more than one embodied agent, where the body is worked on by another or by a team of embodied agents. RBTs are both techniques of the body and for the body. In relation to the former, these include techniques performed by the body and involving a type of knowledge and comprehension comprised entirely in embodied competence, under the threshold of language and consciousness. In relation to the latter, RBTs are techniques that alter and maintain the body in particular ways (Crossley, 2004, 2005, p. 10; Spencer, 2009). RBTs are ordered in ensembles as collections of body techniques that are practiced together for a standard objective. They are intentional insofar that RBTs can be transformed for manifold purposes (Crossley, 2005). Applied to CORs attending CTP, they too are shaped through a process of “modify[ing] their body by working it” (Crossley, 2004, p. 38). CORs learning fitness at CTP engage in *reflexive embodiment*, they use fitness to mould and be moulded. CORs’ ability to be successful in the reflexive body techniques required for physical fitness expectations at CTP (e.g., weightlifting, gym-going, self-defence), is shaped by their past lived-through experiences, innovations, and learning experiences—each shaping present and future body-modification (Crossley, 2001, 2004, 2006).

In this vein, Crossley (2001, p. 83) presents a phenomenological concept of habitus, which is an agent’s “active residue or sediment” of their past habits within the present, actively working to help shape perceptions, thoughts, and actions—influencing their ability to mould social practice regularly. In the phenomenological tradition, habitus is a lived-through structure-in-process, incessantly expanding due to an agent’s or group’s interactions with others and their physical environment (see Spencer, 2009). In the phenomenological approach to habitus, habitus is the product of the power and pre-reflective tendency of the body-subject to habituate and as such, maintain structures of behaviour *and* experience that has proven useful (Crossley, 2004, pp. 39–40; Merleau-Ponty, 2002; Ostrow, 1990). Habitus varies across groups, cultures, and societies, manifesting the corresponding membership (Durkheim, 1995). Habitus materialize into techniques – body techniques and RBTs – that reflect bodily schemas that are learned vis a vis imitation and repetition passed down through traditions and disciplines.

In relation to his study of circuit training, Crossley (2004, p. 39) writes for people to participate in circuit training, agents must know “in an embodied and practical way, how to do it, and they must be disposed to do so.” This reveals a phenomenological schema where CORs come from all walks of life and must know how to exercise at CTP to reproduce body modification (i.e., become fit). Conclusively, CORs may not know how to “work out.” Here, we propose CORs rely on their existing habitus to engage in the reflexive body techniques to be successful in their body project (Crossley, 2004; Giddens, 1991). CORs dispositions and competence of gym-going (habitus) combines with their individual locations within the field, which may continue to shape their perceptions, motivations, and actions towards the RBTs required for correctional work (Crossley, 2001). Therefore, CORs past experiences will affect the “competent pursuit of specified goals” (i.e., using CTP to get in shape) based on this past lived-through experience (Crossley, 2001, p. 84).

Without CSC having physical fitness requirements for occupational entry, CSC may attract candidates with limited “gym-going” experience. Intrinsic motivations (e.g., nature to help prisoners, protection of society, experience with criminal justice work, being a team member) and extrinsic motivations (e.g., living close to the institution, low costs of correctional training, lack of funds, job stability) may supersede as motivations for occupational entry, with little consideration for how fitness falls/fits within correctional work (Britton, 1995; Jurik, 1985; Poklek, 2015; Ricciardelli & Martin, 2017; Ricciardelli, Matthews, et al., 2021; Towns & Ricciardelli, 2023).

Yet to be empirically researched, and as unpacked in the current article, is how the CTP exposes CORs to fitness expectations and physically demanding training exercises with little examination of the tension between the lack of occupational fitness testing and the physically demanding components of correctional work. Our specific attention is directed at the types of physical fitness practiced at CTP, thus valued, and how these fitness practices (i.e., if at all) prepare COs for prison work. Doing so begins a conversation about how the CTP meets fitness needs and if these physical practices are carried over when deployed to a penitentiary. The CO identity is, at least in part, tied to being able to hold one’s own and provide support to colleagues if/when an incident or adversity arises. In this way, being or becoming in shape and introducing a gym going mentality is centralized in CTP and encouraged through organizational anticipatory socialization (see Ricciardelli, 2021). Thus, in the current article, we elucidate how physical fitness and the COR, later CO, identities are intertwined to reproduce a correctional habitus and thus culture of health to ensure safety.

## Correctional work

Existing literature regarding physical fitness in prisons focuses on how physical fitness can ease the experience of incarceration for prison residents by positively affecting moods (Battagalia et al., 2014). Pastor and Cucci (2018, p. 32) highlight how disorders prevalent to incarcerated people in Peru (i.e., depression and anxiety disorders, chronic pulmonary disease, hypertension, and diabetes) can be avoided with increasing fitness resources as a “first line measure.” Battagalia et al. (2014) sampled 64 participants and randomly assigned them across three groups: cardiovascular plus resistance training (CRT), high intensity strength training (HIST), and no exercise. Echoing Pastor and Cucci’s (2018) results, Battagalia et al. (2014) found prison residents exposed to exercise had decreased symptoms of depression in comparison to the control group, with improvements on general/social anxiety and hostility scale scores (Battaglia et al., 2015). Nelson et al. (2006) found, in a pilot study of 105 adult male prisoners that physical activity produced positive mental and physical effects in prisoner rehabilitation when they had substance abuse or behavioural problems. Participants’ symptoms of depression, anxiety, and stress decreased (Nelson et al., 2006, p. 281). With 75% of participants reporting reduced symptoms of anxiety, depression, and stress, they purport how time spent doing productive activities during incarceration may lead to more pro-social behaviour during reintegration.

Researchers also highlight how correctional work subjects prison staff to various types of mental health complications with limited investigation into how similar fitness results could be produced with correctional staff. Largely attributable to a lack of understanding of fitness in correctional work, COs in Ontario experience mental health disorders at an increased prevalence in comparison to other public safety personnel (PSP), with a prevalence of PTSD at 30.7%, Major Depressive Disorder at 37%, General Anxiety Disorder at 30.5%, and 58.2% with at least one mental health disorder (Carleton et al., 2020). In a national study of provincial and territorial correctional workers (CWs), Ricciardelli et al. (2024) found that 58% of CWs (i.e., governance, administration/management, youth CO, program officer, and CO) screened positive for at least one mental disorder, with screening prevalence higher than findings from Carleton et al. (2018) mixed jurisdictional CW sample (i.e., 54.6%) and significantly higher than the screening prevalence of the Canadian general population at 10.1% (Statistics Canada, 2020). Carleton et al. (2018) and Ricciardelli et al. (2024) highlight the consistent relationship between years of service and the increased risk of screening positive for a mental disorder – highlighting the cumulative impact correctional work can cause on CWs. Additionally, 59% of COs from Carleton et al. (2020) Ontario sample screened positive for one or more mental health disorders – prevalence compounded by work-life conflict (i.e., lack of time with family, difficulty decompressing, strain due to emotional/compassion fatigue) (Higgins et al., 2021; Johnston et al., 2022; Lambert et al., 2002) and operational stressors (i.e., poor leadership, interpersonal conflict, CO gossip) (Arnold, 2017; Cassiano & Ricciardelli, 2023; Denhof et al., 2014; Norman & Ricciardelli, 2021). Considering the value of fitness, CSC CORs already feel a need to “prove themselves” to other colleagues based on their ability to protect each other when in distress. CO performance during crisis (i.e., based on a desire for acceptance) can be difficult for new officers (see Carbonell & Ricciardelli, 2023), which showcases the need for physical competency for occupational performances. Revealing how the physical components of correctional work are socially demanding (performance tied to acceptance) and occupationally demanding (being reliable during crisis situations).

Body image, self-presentation and correctional work are also connected to one another, considering traits associated with physical fitness (i.e., muscularity, strength, height) warranted respect from prison residents. Results from Ricciardelli and Gazso (2013, p. 110) research suggest COs who lacked height or were “shorter than average” reported making up for height in other areas such as strength, speed, and muscularity, some even started bodybuilding. Similarly, COs spoke of their disrespect for officers who were overweight or less physically competent (i.e., some who had “let themselves go”), arguing they threaten fellow COs’ safety. Suggesting physical fitness, the ability for COs to “handle their own” and to “prove themselves,” is both a social and occupational preference COs value (Carbonell & Ricciardelli, 2023; Ricciardelli & Gazso, 2013, p. 110). Thus, both prison residents and co-workers reinforce to COs a need to be physically fit or face social repercussions (i.e., a lack of respect from fellow COs). Existing literature suggests the “pains of employment” push COs and people incarcerated to share the burdens of prison life (i.e., pains of incarceration) based on psycho (i.e., symptoms of anxiety, depression), social (i.e., separation from loved one’s), and biological (i.e., exposure to infections and airborne diseases) variables (Sykes, 1958; Turner et al., 2023). Here, however, we ask if CTP provides CORs with the tools to be physically fit, and if CORs continue practicing physical fitness once they leave the CTP?

### Physical fitness in provincial and territorial correctional services

Federal CORs do not complete a physical testing requirement when applying to CSC yet are exposed to fitness expectations at CTP. Despite many provincial and territorial correctional services in Canada requiring occupational fitness testing requirements, this fails to permeate at CSC (see Table 1, which shows what jurisdictions require, and which test, fitness).

**Table 1.** Provincial corrections occupational fitness requirements during recruitment.

Province	Testing Requirements
British Columbia Corrections	COPAT
Alberta Correctional Services Division	COPAT
Saskatchewan Corrections	COPAT
Manitoba Correctional Services Division	No Testing “2 Physical Fitness Standards”
Ontario Correctional Services	FITCO
Gouvernement du Québec Services correctionnels	Techniques de contrôle physique—Services correctionnels
New Brunswick Department of Public Safety	PARE
Nova Scotia Department of Justice	COPAT
Prince Edward Island (PEI) Community and Correctional Services Division	No Testing
Newfoundland and Labrador Justice and Public Safety	COPAT
Yukon Department of Justice	COPAT
Northwest Territories Department of Justice Corrections Services	No Testing
Nunavut Department of Corrections	No Testing

Evidenced in Table 1, most jurisdictions rely on the Correctional Officer Physical Abilities Test (COPAT), a timed physical abilities test intended to replicate the physical challenges recruits will face in the correctional environment. The COPAT consists of three circuits – all to be completed within 3:20 minutes – a cardiovascular component (i.e., Station 1 & 2 stair running and running 50 ft. with turns), resistance component (i.e., push and pull station at 70 lbs), and, a weight and carry finish (i.e., pulling a 70lbs mannequin) (see Government of Saskatchewan, Nova Scotia, and British Columbia website for further COPAT breakdown). In Ontario, they developed the Fitness Test for Correctional Officer Applicants (FITCO) which consists of a three-part “cell search, an emergency response circuit (ERC), and an assessment of aerobic fitness using the Leger 20-metre shuttle run” (Jamnik et al., 2010, p. 72). Jamnik et al. (2010, p. 72) identified how COs found the first two components of the FITCO, including the cell searching component, to be “relatively straightforward” and still the most important component (i.e., stressing the need for CO mobility, range of motion, and strength to be tested before tenure). They suggest the FITCO performance standards “had adverse impacts on female applicants to the position of CO” as the FITCO pass rate for the 56 women in their study was 28.6% (Jamnik et al., 2010, p. 80). In comparison to the 72.7% pass rate for 22 men, suggesting an adverse impact on female applicants (that can be mitigated through the 6-week FITCO-specific training program) (Jamnik et al., 2010).

Conversely, other jurisdictions like Manitoba, Prince Edward Island, Northwest Territories and Nunavut have no physical testing requirements, instead relying on potential recruits having strong fitness capabilities when applying. Provinces, like Quebec, have their own specialized Techniques de contrôle physique, or, like New Brunswick, rely on testing requirements used in policing (i.e., PARE). Thus, ambiguity exists, despite evidence stressing the need for a physically fit labour force (Jamnik, Thomas, Shaw, et al., 2010).

## Methods

To examine how physical fitness is interpreted at CTP, the current study draws on interview data from a longitudinal study of federal COs in Canada. The broader project, entitled “Canadian Correctional Workers’ Well-being, Organizations, Roles and Knowledge” (referred to as CCWORK; Ricciardelli, Andres, et al., 2021) examines the longitudinal mental health and wellness (i.e., its changing context across tenure) of federal COs in Canada. CCWORK collects qualitative (i.e., semi-structured interviews), clinical (i.e., mental health assessment), and quantitative (i.e., surveys) data from COs when they are recruited onto the project (i.e., baseline interviews) and annually thereafter through follow-up waves (Ricciardelli, Andres, et al., 2021). The current study relies on the qualitative data with CORs during their time at the CTP and interview questions probed into officers’ experiences of correctional work; perceptions of correctional training, prison residents, and co-workers; work-life balance; exposure to potentially psychologically traumatic events; correctional policies; and health and wellness. To understand how CORs practice and experience fitness, we analysed transcripts at baseline that arose from discussions about occupational experiences like the stressors involved in the CTP, their perceptions of CO responsibilities and policies, and personal stressors. Recruitment of participants occurs in collaboration with CSC, allowing the Principal Investigator to advertise the study to CORs at CTP (approximately 350–700 participants per year). The scheduling of interviews occurs thereafter, with the *n* = 41 transcripts used for the current study collected virtually during the COVID-19 pandemic and in person from March, 2020 and post May, 2023. CSC continues to support data collection permitting COs to be interviewed (in person or over the phone) during paid work-time. For participants who prefer conducting interviews in French, the principal investigator works with a French speaking team who assists with interviewing and transcription to English thereafter. Despite CSC involvement, participation remains voluntary, CSC cannot access raw data to ensure participant confidentiality. Exclusion criteria included any participant who did not speak about personal or physical fitness in relation to CTP. Participants who did not complete CTP were removed from the study. All interviews are audio-recorded and lasted between 45 and 90 minutes. With each transcript undergoing an initial round of coding using a semi grounded codebook, where primary codes (or nodes, QSR NVivo 2021) are grouped thematically (i.e., inclusive but mutually exclusive codes) and each code represents a specific theme. A second round of applied focused coding begins following a deductive approach, since we had read previous empirical research, to use the lens of physical fitness in public safety work (see Charmaz, 2014; Glaser & Strauss, 2017; Ricciardelli et al., 2011). The current study pulled out codes relating to occupational and personal physical fitness as described by CORs at baseline. Thus, the analysis is a synthesis of emergent patterns from CORs experiences, unpacking potential causal factors of fitness at the CTP.

All participants reported they completed high school (i.e., five stated the diploma was their highest degree), with 19 completing a college program. Six had started a post-secondary program, three were part of the trades prior to corrections, and four completed a university degree. About half identified as male (*n* = 24), with 17 identifying as female, and 10 participants reported having children. Of the *n* = 41 participants, 33 identified as white, four as Indigenous, and the remaining four were grouped as racialized (i.e., Black, South Asian, Arabic). In total, 16 participants were single and never married, two engaged, eight were married, eight common law and seven separated or divorced. To ensure confidentiality and anonymity, interviewees are assigned participant numbers (e.g., P109; P656) not pseudonyms to avoid implicit bias (e.g., racialized or gendered interpretation of names). We edited excerpts for grammar and readability by removing speech fillers or repeated words. Approval was received from Memorial University of Newfoundland’s Health Research Ethics Board (HREB; File No. 20190481) prior to data collection and interpretation. The current study is not without limitations considering the focus on occupational and personal fitness of CORs refrained from asking how fitness programs should be improved or what types of exercises should be taught. Similarly, the findings fail to understand if the lessons learned from CTP carry over to occupational tenure at a penitentiary. Moreover, the current study is also limited in its understanding of how fitness positively or negatively influences COR mental health, including a lack of questions geared towards fitness as a protective factor from mental health problems, voids we invite future researchers to fill.

## Results

We analysed how CORs interpreted and experienced physical fitness at CTP unpacking three central emergent themes: i) how CORs interpreted physical fitness during CTP, ii) how CORs believe physical fitness is tied to safety, and iii) how fitness is enforced/reinforced at the CTP.

### COR interpretations: “I find it easy”

Fitness at the CTP is a multi-pronged approach, with fitness classes such as “high intensity cardio” or “we have fitness classes here and there”, P126, and expectations that CORs engage in 40 min of exercise outside of standard training hours, which is tracked in a log book alongside their caloric consumption (i.e., meals and beverages). Despite CSC promoting and advocating for recruits to be physically fit, P386 highlights how these classes are seemingly unavailable for recruits because they occur “once every two or three weeks”, stressing the need for CORs to, as they are obliged, work out on their own time after structured learning endeavours. Classes that are available were described as “a bit easy” (P120) or “I expected it to be a bit harder” (P130). CTP often fell short in meeting the expectations of CORs who anticipated more challenging classes (i.e., like a military boot camp with push-ups, sit-ups, 5 km runs) or wanted fitness to be highly prioritized. P655, when asked about the least effective component of the CTP, made reference to physical fitness not meeting expectations because of the structure: “there’s really not a lot going on for physical health.” Thus, CORs are expected to engage in 40 min of exercise and log their workouts in a witnessed (i.e., colleagues must attest to the completion of physical activity) log book which is checked infrequently by CTP trainers, delegating to what was described as “the honour system.” P384 explicates how CSC “tries to reiterate and reinforce it but they don’t have a lot here with the fitness. I mean there’s not a lot of fitness classes, a lot of our classes are physical”, showcasing how CTP can be physically taxing through other requirements such as self-defence or restraint training. For instance, P120 argued fitness classes are “pretty easy” but concurrently reports feeling “exhausted” during the self-defence courses at the CTP: “The self-defence, like always getting taken down to the mat and getting up and stuff is exhausting, like we just did a bunch of that today and I am really sore cause it’s different little muscles and stuff but everything else I find pretty easy.” This illustrates how training partners and the “honour system” vis a vis other CORs ensures fitness is being maintained and stresses how RBTs involve the body being worked on by another or by a team of embodied agents in the pursuit of CTP completion (Crossley, 2005). Similarly, the variation in fitness levels across respondents showcases varied habitus of recruits, where CORs come from all walks of life, with varying levels of fitness and knowledge needed to reproduce body modification (i.e., become fit). P181 shares the sentiment that “everything else is easy” but the self-defence components of CTP (i.e., wrestling, doing “back-rolls”, takedowns) are physically harder. Showcasing how the diversity in habitus as lived-through structure-in-processes across CORs positions some to perform better at certain CTP components than others. Being sore from the physically demanding and mandated requirements of the CTP impacts CORs motivations to engage in physical fitness independently, as some CORs believe that gym time can be supplemented during self-defence class. Perhaps, as ethnographic experience supports, the class is not draining in itself, but the length of the class – three hours – creates exhaustion. RBTs are both techniques of the body and for the body in preparation of correctional work.

However, each COR brings various levels of physical fitness depending on previous occupational experience – some who are very fit and some with no traditional “gym-going” experience (Crossley, 2006). For example, P120 describes existing injuries coming into the CTP that inhibited their ability to exercise, stating “the only thing I really enjoy is lifting weights.” Or, the experience of P372 who takes heart medication to keep their heart rate down, meaning they “defiantly can’t do the physical fitness stuff that I would like to do or that I’m used to doing.” Previous tacit knowledge about weightlifting or gym equipment was not shared across all CORs, with some self-identifying as fit but have little experience going to traditional fitness clubs to exercise (while others recognized a lack of fitness). Similarly, some CORs identified a unique struggle of engaging in fitness because of a lack of prior knowledge in how to operate gym equipment. P129 said:

… so I’ve been struggling a lot with that plus the physical aspect. I can go to the gym I can do physical activity. I’ve never had to go to a gym before cause all my jobs have been physically active like my last job for the six years it was walking to a building hold 80 pounds over my shoulder doing stairs. Just laying stuff down and going back and doing that for 10 hours straight. But then when coming out here it’s a very much we need you to go to the gym for 40 minutes a day so it’s like “alright I don’t know what to do at a gym”.

Although CSC wants physically fit CORs, the lack of physical fitness classes offered to recruits at the CTP challenges recruits’ ability to use CTP to “get in shape”, as more emphasis is placed on recruits engaging in physical fitness on their own time. Similarly, P129 who felt like an “idiot” at a private fitness club doing their 40 minutes, describes “getting on the treadmill for the first time in forever it’s like how do I operate this again. I literally stood there for 2 or 3 minutes after I had my movie playing going how do start this.” Suggesting a seeming paradox, CORs describe becoming physically fit at CTP as the “the whole point” P322, – ethnographically, Ricciardelli (2022) watched bodies change over the course of CTP – considering the training offered is “giving you that time to work up your endurance and work up your physicality for sure” but yet, CORs felt fitness to either be too easy or preventing their engagement (e.g., not knowing how to operate gym equipment). With varying levels of fitness, means varying “phenomenon[s] of habit” that Merleau-Ponty (2002, p. 167) suggests is what prompts us to adjust our notions of “understand” (i.e., to be fit) and our notion of the body (i.e., the RBT needed to so). Within this phenomenon of habit, habit is what helps us to experience the harmony between the intention and the performance. Thus, as P322 highlights above, the intention of CTP (or “the whole point”) is the embodied experience where CORs can improve and change fitness levels in preparation for correctional work – thus moulding CORs for the demands of the correctional world to “seamlessly weave their way through space” (Hogeveen, 2013). This reinforces the COR habitus that is incessantly expanding due to interactions with specific other recruits or the broader group and the physical environment of CTP. Thus, CTP should provide CORs with most of the tools to navigate correctional work, including physical fitness.

Although some CORs have limited gym going experience, the comraderies established as a cohort at CTP encourages CORs to, if open to it, teach one another how to perform certain exercises or use equipment at the gym to make sure fitness is practiced. CORs self-identifying as fit spoke about helping unfit CORs. P114 said “it’s not my job to say, so I just try and help him and push him,” while P129 spoke of a fellow COR helping him turn a treadmill and “set it up to what you want it at.” However, despite P129’s experience, he felt “the instructors, are not so much [helpful teaching CORs how to work out], they just said ‘yeah you got to do 45 minutes.’”

Similarly, CORs problematized individualized approach to fitness because “I don’t find there’s much time to do fitness” as the academic and scholarly classes happening concurrently with fitness expectations, can take priority based on subjective skill levels. P652 highlights how fitness can be undervalued when recruits are “so busy with assignments and studying and stressing about passing everything that I haven’t been taking time to work out on a consistent basis.” Leading CORs yearning for more structured fitness classes, partially because not all CORs enter CTP with an understanding of how to workout at a fitness club, nor, and understanding of how to use gym equipment effectively to engage in the body modification necessary to get in shape (see Crossley, 2004).

The reported stress-inducing obligations of CTP are compounded by the perceived lack of physical fitness equipment available for CORs to use at CTP, often leading them to travel to the nearest fitness facility to access equipment (i.e., another source of stress). P440 described CTP as not having a typical gym with equipment similar to a private fitness club (e.g., Planet Fitness, Goodlife Fitness, Fit4Less) despite some CORs being misinformed about equipment at CTP. P151 explains:

I thought the way they run their fitness program would be a lot different, From what they explained it to me they made it sound like they had a whole gym worth of stuff, like workout room in the gym, they didn’t. It’s kind of more on our own to find our own stuff. And, yes we should be able to do it but just having to go out and since everything is far away, kind of restricts it in that way.

P151 felt CSC was ingenuine and made them believe CTP would have fitness equipment similar to private fitness clubs, but was surprised to learn facilities had only “kettle bells and all that kind of stuff.” In response, some CORs joined private gyms – despite limited funding at CTP. Using private facilities was a plausible source of stress due to inaccessibility (e.g., “the closest one is like 15 20 minutes away” (P151). P251 echoed these stressors, discussing, how physical fitness expectations offsets normal work/night-time routines when “I have to stay here for my meal then go out and drive twenty minutes across town to go to the gym.” P90 was also “amazed” CTP did not have a gym, that reinforced the fitness paradox: “we’re supposed to becoming physically fit” but “at the same time the facilities don’t exist for us to be training the way we should be.” Thus, the “whole point”, or at least a major component, of CTP is challenged if recruits struggle to accessibly and effectively work out. In theory, we can use Merleau-Ponty’s (2002) phenomenological approach to habitus to understand how the CTP attempts to produce understanding bodies and habits needed for CORs to navigate the correctional world with ease. But yet, the CORs from our sample are fully immersed in the space that would allow them to learn the reflexive body techniques (RBTs) necessary to practice physical fitness, but, concurrently, struggle with a lack of opportunity to learn the corporeal schemas necessary to grasp its significance. Despite participants wanting CTP to teach them the habits and RBTs to be physically fit, there is a lack of opportunity or intellectual engagement necessary for them to do so. Said otherwise, a COR may know that incident codes require cardiovascular endurance, but, if they lack the knowledge to properly use the treadmill, the movement will never be fully assimilated into their bodies and thus not activated within federal penitentiaries (see Crossley, 2001, 2004; Hogeveen, 2013; Merleau-Ponty, 2002).

### Perceptions of fitness: “would I want him watching my six?”

CORs enter CTP with historical trajectories, each informing experiences and learning. CTP directly affects how CORs perceive the physical fitness abilities of fellow recruits and how CORs believe fitness abilities will affect their safety at federal penitentiaries. The safety component of physical fitness caused many CORs stress, often raising concerns about their colleagues’ ability to respond to incidents within penitentiaries in a timely manner. While at CTP, recruits are exposed to their colleagues’ varying levels of fitness for the first time, often informing their interpretations of officer safety. We asked CORs about their interpretations of their colleagues’ fitness levels, to which P107 responded how recruits had varying capabilities, some lacking in fitness. P107 then raises concerns about “watching my six” (i.e., watching my back) arguing there’s “not a fucking chance in hell” given the lack of physical abilities that a particular colleague would be able to provide protection:

The guy couldn’t walk up a flight of stairs so what do you think he’s going to able to do? Do you think he’s going to be able to respond to an emergency situation or he’s got a bunch of OC [oleoresin capsicum spray; pepper spray] in his face do you think tussle with a guy and wrestle a guy to the ground do you think he’s going to be able pull down an officer off a unit no.

Although de-escalation and negotiation are valued more for COs in comparison to physical intimidation or force (Farkas & Manning, 1997), P107 highlights how officers must still be able (and ready) to perform physically demanding tasks to keep prison residents and fellow officers safe. Having the backs of fellow officers was valued by CORs among their recruits (echoed by Ricciardelli & Gazso, 2013). Unfit CORs left colleagues concerned about their ability to remain safe during crisis or critical incidents. P114, for example, does not “want to be stuck range with him [unfit colleague] if shit hits the fan” while P121 stated people at CTP “can’t do the most basic things” but at the same time “you are working with this person like this person is supposed to have your back and it may not work out too well if they keep going in the route that they are going with their health.” Thus, beyond fit CORs, they call to action to other CORs to improve their fitness. P385 continues to explain how fitness should be reciprocal between colleagues, thus motivating each other to increase their stamina and abilities. Some CORs felt those who truly struggle with fitness may self-select out of CTP (i.e., “they are not here anymore” or “they went home”), suggesting it should not be resolved in such manners, instead there should be criteria tied to fitness (as discussed below). Describing an ideal officer, participants said:

They’re put together and they’re present. They’re ready to go. They don’t have to be told to stop talking or things like that. A lot of it too is physical. A lot of it in this job is physical handling and stuff like that but as well it’s talking to people, confidence, or a small person – there’s nothing wrong with that but you can’t be out of shape. I think that’s a negative. P28

To me it’s just like it overall confidence that has to become and just overall confidence that I see in these people, age as well and I guess would be just be physical there physical status. P37

In the first excerpt, P28 describes an ideal officer as present, willing to help, and confident with physical handling capabilities. In the second excerpt, P37 too reflects on the need for COs to be confident and fit, if there is to be a sense of safety. Competence levels in fitness (e.g., size, physicality, physical handling) shape security:

If someone is more physically fit and comes along rather than somebody that’s physically unfit I [am] going feel a lot safer with the person who’s really fit right especially if I’m on the complete other side of the institution during a night shift and something really bad happens. (P79)

Despite federally employed COs in Canada needing to graduate the CTP before occupational tenure, recruits like P121 highlight how colleagues fail to possess the corporeal schema’s needed, described to us as “the most basic things”, but are concurrently responsible for the safety and security of both prison residents and staff. As per P79s quote, CORs perceive increased safety when supported by fit colleagues, who in an embodied way, can use the adaptability of habit (i.e., physical fitness) as a “general form” to help resolve varying occupational demands (i.e., incident codes, staff assaults, use-of-force) (Merleau-Ponty, 2002). Comments from P28 support Crossley’s (2006) finding that physical fitness influences the social capital/social life for gym goers, however, not all CORs know the RBTs needed, possess the habitus, or are granted the physical capital (i.e., equipment) needed to produce the corporeal schema of physical fitness. Thus, a lack of fitness not only influences perceptions of safety but may also worsen the social interactions between colleagues (see Carbonell & Ricciardelli, 2023), which Cassiano and Ricciardelli (2023) argue is a major source of stress in Canadian correctional service work.

### Enforcement of fitness: “some people never workout”

CSC lacks a fitness requirement at occupational entry. Thus, recruits are exposed to physical fitness requirements (i.e., some for the first time) upon arrival at CTP. This led some, like P284, to perceive fitness not to be a CSC priority, elaborating “they [CSC] don’t have any fitness training to get in, which I don’t think is a very useful, but they’re trying to get more people in the door, so I think they’re just kinda turning a blind eye to people who are not physically fit coming into the program.” Moreover, without a testing requirement, accepted CORs could experience increased strain tied to their own physical status. For example, P322 highlights the “self” significance relating to how they were once in very good shape but are not anymore (e.g., life circumstances, age, personal issues; cf. Crossley, 2006) which resulted in CTP being very physically demanding—they were not prepared for the physical component of CTP: “I mean I was doing really really well the first week and then the last two weeks I’ve just been so freaking sore and tired that I haven’t been able to keep up with my gym time which really has been disappointing because I was really enjoying it” (P322). P122, too, was “shocked” about the lack of testing requirement and argued the COPAT (i.e., Correctional Officer Physical Abilities Test, used in some provincial corrections) should still be mandatory for CSC. P122 said:

I’ve seen already being here for one month and I’ve seen people that haven’t gone to the gym, I’ve seen people that when they do go to the gym they say “I walk I don’t see what there’s no actual set standards as to what you need to pass.” RCMP, when you go there, there’s COPAT and you have to pass it. You get two chances, if not off you go and it’s not just for your health but it’s for the safety of other officers.

P122 argues the physical testing is an officer safety provision. Echoing how CORs are exposed to overlapping fitness expectations (i.e., physical fitness classes, 40 min of personal exercise, and self-defence), the requirement can be overwhelming for unfit people, leaving CORs to reduce their external fitness practices, like gym-going. Moreover, the lack of fitness enforcement, beyond the infrequent logbook checks by CTP trainers, creates reservations about how CSC values COR fitness: “I’ve been told I’m in decent shape and I want to get stronger obviously. That’s one thing I’m working on here. Some people never work out, ever. Except in the classes” (P28). The lack of enforcement, in combination with the little gym-going experience for some, results in “people not participating as much” (P128), and even fitness mindsets that add little value (e.g., “I’m at the gym what does it matter what I’m doing?” (P129).

One suggestion to increase the emphasis on fitness was to make fitness more valued by being a “strikeable” requirement. To explain, CTP has a three strike system, where inadequate test scores (i.e., under 70%) or unethical behaviours can result in CORs accumulating strikes. Although the strike system can be a source of stress (Ricciardelli, 2021), some CORs felt fitness should be a strikable requirement beyond the “honour system.” P383 explains:

definitely 100% I think that I would change that I would definitely make sure that there is a strikeable on fitness I mean it doesn’t have to be a huge one it doesn’t have to be like oh you got to be able to do chin ups and stuff but making sure your being fit because you have to be the person who’s going to run down that frigging hall way and be able to help the other officers getting attacked and if you were going sitting there dying on the spot like you’re not going to be able to help anyone so I don’t know I feel like fitness needs to have a little bit more weight

Although fitness is enforced based on CORs witnessing log book checks (i.e., CORs must attest to the completion of physical activity), P383’s words suggest CORs want fitness to carry harsher enforcements, despite concerns that the strike system was already a source of stress for some. Thus, the perceived lack of physical fitness enforcement showcases a habitus that is the product of power and pre-reflective tendency of the body-subject to habituate and as such, maintain structures of behaviour and experience (i.e., being fit versus unfit moving into occupational tenure) (Crossley, 2004, pp. 39–40; Merleau-Ponty, 2002; Ostrow, 1990). A lack of fitness classes or strength and conditioning programs for CORs results in an inability for an embodied habitus – that could materialize into techniques for bodily schemas to be learned vis a vis imitation and repetitions – to be developed at CTP before CORs enter federal penitentiaries (Crossley, 2004, 2005, p. 10; Spencer, 2009). The bodies of CORs and COs must be able to reproduce the RBTs in real time to support the safety of prison residents and prison staff, and failing to do so could lead to breaches in institutional and personal security.

## Discussion

CORs outlined how physical fitness was necessary for correctional work, for officer safety and preservation of life. Echoing findings of Ricciardelli and Gazso (2013), CORs value physical prowess as an officer safety provision, and, as per Carbonell and Ricciardelli (2023), physical fitness is also a social component where mitigation of violence during critical incidents (i.e., based on physical abilities) is a way for CORs to be socially accepted by having each other’s backs (i.e., desire for acceptance). Given CSC is without a physical testing requirement for entry, recruits from a variety of occupational experiences can apply (i.e., lived through structure in process; Crossley, 2004). Inadequate testing left some to suggest that although it may increase the number of recruits, safety too must be a consideration. Some recruits had no previous “gym-going” experience, making it incredibly difficult to engage in the reflexive body techniques (RBTs) necessary to “get in shape” (i.e., body modification). The embodied performance and moral significance of using CTP to get “in shape” (Crossley, 2006), was described by some as the “whole point” of the training program—an opportunity for a lifestyle change for those who were not fit. Our findings suggest that although CORs attribute fitness with the CTP, some have no prior gym-going experience (e.g., not knowing how to use gym equipment or to engage in the reflexive body techniques necessary to become fit) which is compounded by the minimal exercise equipment at CTP. The corporeal schema of physical fitness requires both an understanding of how to exercise and the embodied ability to engage in the RBTs needed to produce body modification oriented towards the active body project. Recruits can learn in the CTP classroom about weightlifting, running on the treadmill for 30 min, and conceptually understand the benefits of callisthenics, but until their body experiences the movements needed to do so, their bodies will never be activated appropriately within the correctional setting. Providing potential insight as to why participants in our sample highlight CTP is physically demanding, because CORs are engaging and practicing self-defence (i.e., some for the first time), and do have fitness classes, but do not provide fitness instruction for gym going, for example. For CSC, CORs are not given *all* the tools necessary to effectively and accessibly engage in fitness: there are no fitness requirement for occupational entry, but there are fitness requirements to be met. Incident codes are a common occurrence within most federal penitentiaries, often requiring COs to run at top speed to ensure the safety of prison staff and residents, but yet, some CORs from our sample highlight a lack of instruction on how to operate gym equipment like treadmills. Considering sprinting is but one of the necessary physical expectations of the CO role, CORs are not taught the reflexive body techniques required to train cardiovascular endurance at the gym, showcasing the lack of fitness requirements despite fitness expectations within federal correctional services. Physical fitness and a physically fit workforce is crucial for the CO role, considering service delivery begins (i.e., first aid, use of force that is also physically demanding) after a physically demanding exercise like sprinting ends.

Although recruits at CTP with more tacit knowledge on gym-going did support fellow CORs in learning the operational nuance of being at the gym – many CORs, given the individualized approach to fitness that CTP prioritizes, felt CORs are underprepared for the physically demanding components of CTP. Many CORs interpret the mandated fitness classes as “easy” and infrequent, not meeting the needs of CORs who want fitness to be more prioritized. However, the fitness classes offered do not teach CORs with no gym-going experience how to effectively and safely exercise at private fitness clubs.

Without direction, beyond 40 min of exercise, some CORs felt lost – not sure how to pass that time but wanting to adhere to the demands of being a recruit. For example, P427 understands the need to be physically competent for correctional work, but doesn’t stress the need for “gym-going”: “to me like is it important absolutely do you have to work out at some point yes. I just walk my dog like an hour a day and I’m up and doing things but I don’t necessarily go to the gym for two hours a day.” Our findings suggest CORs who possess the RBTs necessary to be fit, are more well perceived within correctional services in comparison to unfit colleagues – potentially increasing perceived levels of job satisfaction among COs. The ideal officer was described in our sample as present, willing to help, and confident with physical handling capabilities. Thus, CORs who possess the habitus to be physically fit are more likely to accumulate the social capital necessary to “fit in” and achieve the desire for acceptance, potentially reducing occupational stresses as COs are more likely to feel supported by colleagues.

Given some recruits found certain components of CTP exhausting, like the self-defence training, and causing muscles to be “sore all the time,” entering CTP unfit or with a lack of gym-going knowledge, can cause adverse consequences (i.e., feeling overwhelmed or stressed). In consequence, some miss gym time (i.e., due to sore muscles or the stress of “strikes”), supplement gym time with self-defence training, or fail to meet expectations of fitness and self-select to leave CTP. Relatedly, the individualized approach to fitness outlined by our participants is also problematic. Forcing CORs to work out who do not have the corporeal schema, nor the embodied habitus to properly execute the RBTs needed for body modification, can lead to injuries. For instance, Crossley (2004, p. 53, 2006) writes in relation to circuit training that agent’s must know when to temporarily cease the usual habit of the activity “at the point at which it hurts or renders them short of breath, activating the acquired perception habit of experiencing the ‘burn’ positively.” CORs who have no “gym-going” experience are at an increased risk of imminent injury considering they have yet to acquire the “mastery of reflexive body techniques” of knowing the difference between the positive or negative “burn.” Creating realities where CORs execute poor form during exercises, worrying about inappropriate injuries rather than “transforming their embodied intentionality” (Crossley, 2004).

Thus, without a fitness test or fitness program for recruits prior to CTP (i.e., similar to the 6-week FITCO-specific training program in Ontario Provincial correctional services; Jamnik et al., 2010) recruits may be underprepared for the physically demanding components of CTP, which may result in self-selection out of the program. Some CORs may be made vulnerable by their lack of fitness related knowledge (using machines, exercise regimes, or not knowing how to operate equipment). Of course, the lack of fitness requirements may increase the pool of potential recruits, the possibility of self-selected discharge may be compromising this benefit, as a recruit with little experience in previous occupations surrounding fitness, or little “gym-going” experience, may struggle to meet expectations of fitness while at CTP, despite potential strengths in other areas of correctional work which are valued (i.e., de-escalation or negotiation) (Farkas & Manning, 1997).

## Conclusion

In conclusion, we recommend CSC reconsider fitness requirements for their recruits, thereby reducing the margins of attracting physically unprepared candidates, some self-identifying in the current study, who may struggle to balance the concurrent expectations at CTP. Relatedly, CSC should consider provisions for future occupational testing requirements similar to initiatives undertaken at the provincial level like the province of Ontario, who developed the Fitness Test for Correctional Officer Applicants (FITCO) consisting of the “cell search, an emergency response circuit (ERC), and an assessment of aerobic fitness using the Leger 20-metre shuttle run” (Jamnik et al., 2010, p. 72). Doing so would allow for an assessment of future recruits’ abilities across job specific tasks (i.e., cell-searching), aerobic capacity to ensure recruits can both timely respond (i.e., sprinting), and then preform potentially demanding job roles like use-of-force or first aid (i.e., requiring endurance) thereafter. However, CSC must be aware of and ensure the creation of a bona fide occupational testing requirement does not adversely affect “female applicants to the position of CO” to ensure all applicants have an equitable opportunity to become a federal CO – a fruitful area of future research (Jamnik et al., 2010, p. 80)

Recognizing the creation of occupational testing for recruitment takes time, an immediate reform CSC may want to consider implementing is a strength and conditioning module at the CTP to help structure the required daily 40 min of physical fitness mandated outside of class. Having professionals teach proper technique and instruction on how to exercise to CORs (e.g., how to use machines, how to engage in proper exercises, how to weightlift) may reduce the potential of inappropriate injuries and address gaps identified by recruits in the current study who desire more knowledge, access and time. Moreover, perhaps investment into more onsite exercise equipment/facilities would help promote CO fitness by making equipment more accessible, considering CORs in the current study who travel to private fitness clubs outline travel time is an additional stressor. An onsite facility, that includes a diversity of fitness equipment like squat racks, smith machines barbells, treadmills, rower machines, yoga mats, stationary bikes, or dumbbells, may reduce barriers to fitness such as lack of previous gym-going experience (i.e., by increasing access), and in combination with strength and conditioning classes, may also increase the likelihood that CORs continue to practice fitness moving into occupational tenure at federal penitentiaries, increase perceived job satisfaction where colleagues feel supported by physically fit co-workers, and allows for fitness to be used as a coping strategy considering the adverse climates COs work in. Establishing onsite facilities also creates a unique opportunity for CSC to provide naturalistic recovery remedies for CORs like cold tubs, sauna’s, red light therapy, or hydromassage machines that may reduce CORs fatigue, a physical barrier described by participants in the current study and increases the likelihood of CORs completing and graduating the CTP rather than self-selecting out due to lack of endurance.
